# Awareness, treatment, and control of hypertension and related factors in adult Iranian population

**DOI:** 10.1186/s12889-020-08831-1

**Published:** 2020-05-12

**Authors:** Mohsen Mirzaei, Masoud Mirzaei, Behnam Bagheri, Ali Dehghani

**Affiliations:** 1 Yazd Cardiovascular Research Center, Shahid Sadoughi University of Medical Sciences, Yazd, Iran; 2 Shahediah Cohort Study, Shahid Sadoughi University of Medical Sciences, Yazd, Iran; 3 Department of Epidemiology and Biostatistics, School of Public Health, Shahid Sadoughi University of Medical Sciences and Health Services, Yazd, Iran

**Keywords:** Hypertension, Awareness, Control, Iran

## Abstract

**Background:**

Hypertension, known as the silent killer, is a major risk factor for cardiovascular disease. Awareness and treatment of hypertension is not appropriate in the world, and this has led to an increase in mortality and morbidity caused by uncontrolled hypertension. This study aims to estimate awareness, treated, and controlled hypertensive and relevant predictors in an adult Iranian population.

**Methods:**

This cross-sectional study was conducted on 10,000 adults aged 20–69 years in Yazd, Iran. They were selected through multi-stage random cluster sampling in 2015–2016. Blood pressure was measured three-time with standard protocol by trained health workers. Those with a positive history of hypertension and using anti-hypertensive drugs, prescribed by a physician, were considered hypertensive. Hypertension was defined as systolic blood pressure ≥ 140 mmHg and/or diastolic BP of ≥90 mmHg. Uncontrolled hypertension was defined in accordance with recommended treatment targets by the Joint National Committee (JNC7). Logistic regression was used to assess the predictors of hypertension awareness, treatment and control.

**Results:**

The prevalence of hypertension was 37.3%, and the prevalence of pre-hypertension was 46.4%. 49.7% of People with hypertension were aware of their disease, and 71.5% of them were using antihypertensive drugs prescribed by physicians. Blood pressure was controlled in 38.9% of the treated patients. In the adjusted model, older age, female sex, and history of diabetes mellitus were positively associated with higher awareness. High physical activity, tobacco smoking, and diabetes are the only predictors of treated high blood pressure. Younger age, female sex, and higher education were determinants of controlled hypertension. Having health insurance was significantly correlated with awareness and control of hypertension.

**Conclusion:**

Hypertension is a public health problem in this population, which is not well controlled. Half of the patients were unaware. Intervention for increased screening coverage is needed. It should plan to raise public awareness about hypertension and improve hypertension control under the supervision of physicians. Implement a family physician program is recommended in the health system.

## Background

High blood pressure is an important risk factor for cardiovascular disease and causes 7.5 million deaths per year (12.8% of all deaths) annually [1]. The global burden of disease study suggests that systolic blood pressure is accountable for the highest proportion of lost years of life due to premature death, with 212 million years lost [2]. The high blood pressure rank in the world increased from the fourth in 1990 to the second in men and first in women in 2017 [3]. The prevalence of hypertension (HTN) in various regions of the world has been reported from 4 to 78%. In the Eastern Mediterranean region, it is on average 29.5% and in Iran 22% [4–6].

Among the known risk factors for non-communicable diseases, hypertension after high Body Mass Index (BMI), unhealthy diet, and high blood glucose is the fourth risk factor, which has increased by 6.7% from 2005 to 2016 [7]. Yazd Healthy Heart Project reported the prevalence of HTN 25.6% in Yazd [8]. The number of people with HTN in low-middle income countries (1.04 billion) is higher than in developed countries (694 million), which shows an increasing trend from 2000 to 2010(7.7%). However, it decreased by 2.6% in high-income countries [9–11].

Despite the high prevalence, studies have shown that in the world, the percentage of unawareness, untreated, and even uncontrolled HTN is significantly high [9]. In general, 50–75% of patients with hypertension do not receive proper treatment [12]. In Iran (2011), 43.2% of patients are aware of their illness, 34.8% of hypertensive persons are treated, and 38.6% of them are controlled which vary across provinces.

In Northern Iran, about one-third of the treated patients have controlled hypertension [13]. In Azar’s cohort, 60% of the participants are aware of their illness, and 68% have controlled blood pressure. In Shiraz, 69% have controlled blood pressure [13–15]. The 2010 study in Yazd showed that the rate of awareness for hypertension was 43.7% of the patients. 77.1% of them were treated, and only 12.4%, who treated, had controlled blood pressure [16]. This difference between the prevalence, awareness, and control of high blood pressure among countries as well as among different regions of a country in other studies is also reported [17]. The most important complication of uncontrolled blood pressure is morbidity and mortality of cardiovascular diseases (51% of stroke and 45% of deaths due to myocardial infarction) [18]. Inappropriate management of HTN can be the result of socioeconomic factors such as poor health literacy, lack of access to health care providers due to lack of centers, or inability to pay for health costs, and so on. Identifying these factors may help to design more effective health interventions. The purpose of this study is to estimate the prevalence of awareness, treatment, and control of hypertension and relevant predicting factors in an adult Iranian population.

## Methods

This study is a cross-sectional analysis of the data from the recruitment phase of the Yazd Health Study (YaHS), which is a population-based longitudinal study designed to determine the prevalence of non-communicable diseases and their risk factors in Yazd Greater Area. The maximum sample size was calculated according to 50% prevalence and significance level of 99%, for all the NCD and their risk factors. The initial sample size was calculated 538. It was corrected based on ten strata of five participants in each age group (20–29, 30–39, 40–49, 50–59, 60–69 years) by each sex in clusters. The design effect of 1.5 was considered, it was predicted that 5% would not respond in the recruitment phase (*n* = 8494) and 15% attrition rate or loss to follow-up was predicted and added to the number which was reached to 9768 in the second wave. Thus, we decided to enroll 10,000 persons in the study. Blocks of urban-rural neighborhoods were considered as clusters. The blocks were randomly selected. According to tossing the cluster random sampling method, 10,000 residents of Yazd (20–69 years old) were selected from 200 clusters in years 2015–2016. Of each age group of ten, five were selected in clusters of 50 (25 men and 25 women). A completed method of study was published elsewhere [19]. Informed consent was given to participate in the study. According to the protocol, the questionnaire is repeated every five years to provide longitudinal information for determining the risk factors for health and the incidence of disease.

The interviewers completed a valid questionnaire and measured anthropometric and blood pressure at a home visit. See Additional file for the YaHS questionnaire (s1). The overall response rate was 98% (*n* = 9800). Demographic characteristics, history of cardiovascular disease, and the relevant risk factors were recorded. Trained people in a sitting position measured physical examination and after rest, using a standard and appropriate cuff size for the participant’s arm [20]. The pressure measurement was carried out three times at five-minute intervals by calibrated Reichter electronic sphygmomanometers (Model N-Champion, Reister GMBH, Germany), which were calibrated regularly. The mean of second and third measurements was recorded as blood pressure and used for analysis. People with the following characteristics were classified hypertensive case: a) Self-reported previous diagnosis of hypertension by the physician, and b) systolic BP ≥140 or diastolic BP ≥90 mmHg according to the Joint National Committee JNC7 classification [21].

Awareness of hypertension was defined as a self-reported previous diagnosis of hypertension by a physician among the participants with hypertension. Unawareness of hypertension was defined by blood pressure ≥ 140/90 mmHg without a prior diagnosis by a physician or the use of any antihypertensive drugs.

The participants who were aware of their hypertension, who answered the question: “When was the last time you referred to a doctor for your high blood pressure?”, “over the past three months,” were categorized in the treated group.

Controlled hypertension was defined for those taking antihypertensive medication for the management of high BP at the time of the interview. It had systolic BP < 140 mmHg and diastolic BP < 90 mmHg. Uncontrolled hypertension was defined following recommended treatment targets of systolic BP ≥140 mmHg and diastolic BP ≥90 mmHg (Including those who were aware). SBP/DBP goals recommended for Specific disease (diabetes mellitus) was < 130/80 mmHg [22].

Physical activity was assessed by the International Physical Activity *Questionnaire* (*IPAQ*), short-form (SF). It examines the intensity of physical activity over the last week for different levels, individually. Metabolic equivalent (MET; multiples of resting energy expenditure) by minutes per week estimated by self-reported duration (in minutes) and number of days for types of activity in the past seven days. Finally, participants were classified into three levels of “low”, “moderate” and “high” physical activity [23]. Body Mass Index (BMI) calculated as weight/height^2^ in kg/m^2^ and was classified to underweight < 18.5, normal = 18.5–24.5, overweight = 25.0–29.9, and obese ≥30.00 [24]. Those who answered “Yes” to the question “Do you smoke cigarettes or hookah?” were considered current smokers [19].

The study was approved by the ethics committee of Shahid Sadoughi University of Medical Science, Yazd, Iran (IR.SSU.MEDICINE.REC.1396.311). The study was explained to all respondents willing to participate. All participants had the right to withdraw from the study at any time. Informed consent was obtained from each participant before data collection. Participants with a new diagnosis of hypertension were advised to refer to their health center or physician for the follow-up.

Descriptive statistics were reported, and age-standardized prevalence rates were calculated using the direct method based on Yazd and Iran population in the national census 2011 [25]. Awareness, treatment, and control of hypertension were presented as percentages. A chi-square test was used for categorical variables to analyze the differences in demographic variables between the groups. Binary logistic regression was fitted. For binary logistic regression, two groups were defined; aware and unaware, treated and untreated and controlled and uncontrolled. It performed to ascertain the effects of age, gender, education, health insurance, place of residence, BMI & physical activity, smoking and history of diabetes mellitus (as independent variables) on the likelihood that participants who are aware of their illness, those who are being treated and whose blood pressure is controlled. Multivariable logistic regression analyses (enter method) were performed to assess the association between dependent (awareness, treated, and controlled hypertension) and independent variables. Crude differences in proportions were presented by using *χ*^2^-tests. Association of independent factors with awareness, treatment and control of hypertension (dependent variables) were reported as odds ratios with 95% confidence intervals (CI) after adjustment. All statistical analyses were performed using SPSS version 16 software. A *p*-value of less than 0.05 was considered statistically significant.

## Results

Of the total respondents, 49.2% of participants were men and 4.1% were from the rural areas; 25.7% had primary or less education; 15.7% of the participants had BSc, MSc. or doctorate degrees; 94.5% of the participants had universal health insurance. Most participants (84%) were married; 68.9% of men and 11.7% of women were employed and 74.4% of women were housewives.

Of the total 9800 participants, 1817 (18.5%) had a history of hypertension, 45.6% of those between 60 and 69 years old. Hypertension was more prevalent in women (21.9 vs. 15.2%, *P* <  0.0001) than men. The age-standardized prevalence of hypertension in this population was 10.5%. age and sex standardized prevalence rates of hypertension was 12.03 according to the national population census (Male: 9.2%, female: 14.2%) [25]. To enable comparison across regions, we used the World Health Organization (WHO) ‘world’ population for age and sex standardization [26]. According to WHO population, the prevalence of hypertension was14.04% (male: 11.6%, female: 16.5%).

The frequency of high blood pressure was higher in people with less education. Hypertension is more common in the indigenous population compared to migrants from other provinces (19.5% vs. 13.1%, *P* <  0.0001). A history of two years or more of hypertension has been reported in 72.4% of patients. 28.8% of patients did not refer to the doctor for the treatment of their high blood pressure for four months or more. Socioeconomic factors and family history of common disease associated with self-reported hypertension in Yazd greater area was shown in Table 1.

**Table 1:** Socioeconomic factors associated with self-reported hypertension in Yazd Greater Area. 2014–2015

	Num.	Percent
Crude prevalence	1817	18.5
Age-Standardized prevalence	10.5	
Sex		
Men	737	15.2
Women	1080	21.9
Age group		
20–29	29	1.5
30–39	79	4.0
40–49	270	13.3
50–59	577	29.5
60–69	861	45.6
Education		
primary school and less	881	34.6
high school	484	17.4
diploma and graduate diploma	323	11.3
BSc & MSc. and doctorate	110	7.3
Migration status		
Yazd native	1374	19.1
From within the Yazd province	228	22.2
From other Iranian provinces	161	13.0
from overseas	29	13.8
Duration of hypertension (years)		
< 1	113	6.6
1–2	356	20.9
3–4	370	21.7
5–6	268	15.7
> = 7	595	35.0
Total	1702	100
When was the last time you visited your doctor?		
< 1 month	570	34.0
1–3 months	625	37.3
4–6 months	219	13.1
6–12 months	115	6.9
> 1 year	147	8.8
Total	1676	100
Having health insurance	1733	97.4
positive Family history of hypertension	1247	72.2
positive Family history of CVD	686	39.8
positive Family history of DM	705	39.0

Overall, half of the adults with hypertension were aware of their disease (49.7%). This proportion increased with age, from 11.9% at the age of 20 to 29 years to 67.0% at the age of 60 to 69 years (*P* <  0.0001). Women were more aware of their disease (*P* <  0.0001). Hypertension was more common in overweight and obese patients, less educated, those with low physical activity, and patients with a positive family history of cardiovascular disease and diabetes (*P* <  0.0001).

The age and sex standardized prevalence of newly diagnosed hypertension (unaware patients) in this population was 16.2%. The mean age of newly diagnosed hypertension cases were nine years lower than known cases (47.4 vs.56.9) (*P* <  0.0001). Most newly diagnosed hypertension patients were male (62.5%), 70.7% of these patients were found to be in the stage I of hypertension. The prevalence of diastolic hypertension was higher in this group compared to systolic hypertension (67.4% vs. 59.5%). The mean systolic blood pressure of this sub-group was slightly lower than known cases (140.3 mmHg vs. 141.9) (*p* = 0.017). However, the mean diastolic blood pressure in this group was higher (93.1 mmHg vs. 85.8) (*P* <  0.0001).

**Fig. 1:**
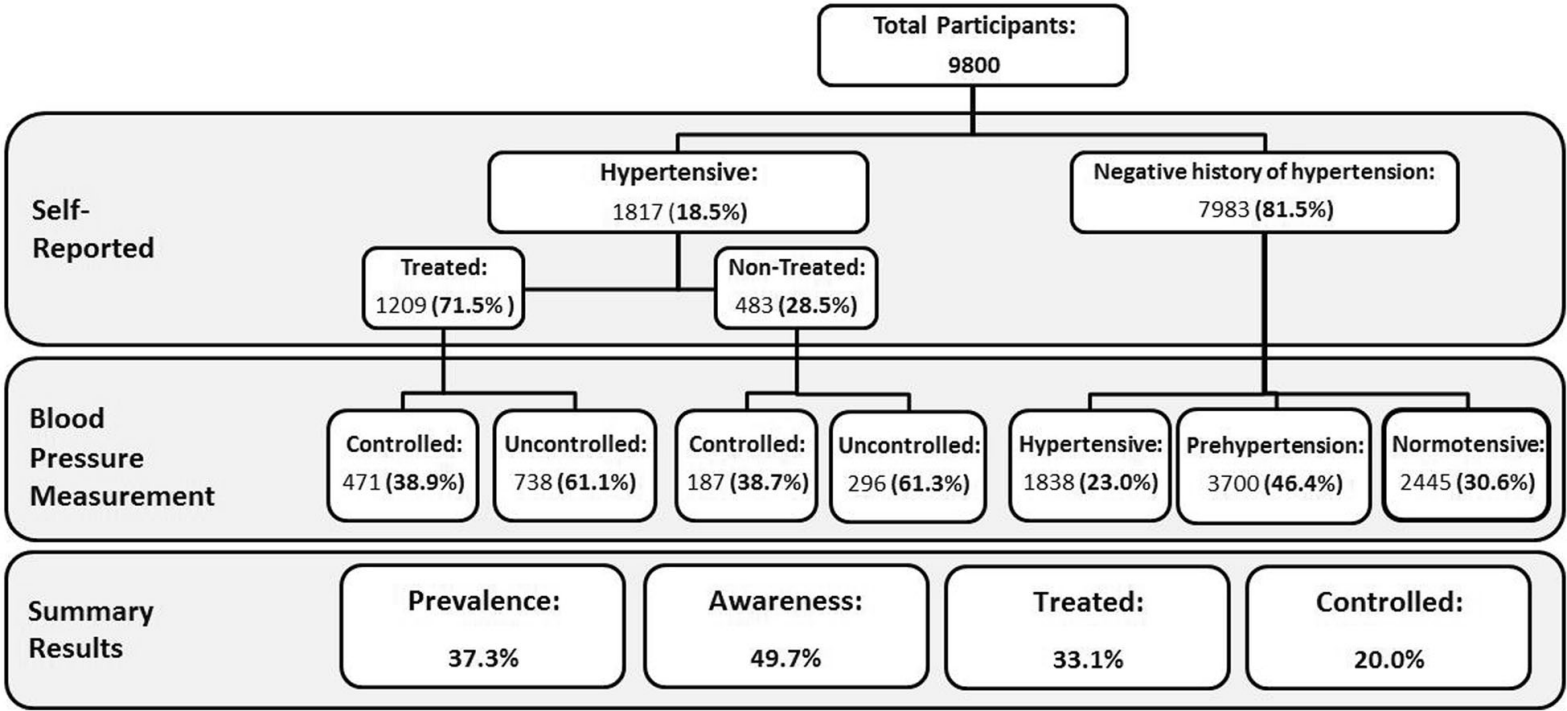
Hypertension, its awareness, and control among adults 20–69 years in Yazd Greater Area- Iran

71.3% of the aware patients (33.0% of all participants with high blood pressure) were referred to physicians during the past 3 months. Younger and more educated people are less likely to go to treatment by physicians, compared to older people (*P* = 0.015) and illiterate (*P* = 0.024). The control of hypertension among males was significantly lower than females; also, uncontrolled hypertension increased with age (*P* <  0.0001). There was no significant difference in terms of the treatment and control of hypertension between those with and without health insurance, physical activity, place of residence, or abnormal BMI (Table 2). Figure 1 shows a summary finding of prevalence, its unawareness and uncontrolled hypertension among adult participants.

**Table 2:** Prevalence of awareness, treatment, and control of hypertension in Yazd adult residents aged 20–69 years (Total hypertensive *n* = 3655)

	Awareness Num. (%)	Treated^a^ Num. (%)	Controlled^a^ Num. (%)
Crude prevalence	1817 (49.7)	1209 (71.5)	731 (40.2)
Age groups			
20–29	29 (11.9)	9 (52.9)	20 (69.0)
30–39	79 (19.2)	37 (56.1)	40 (50.6)
40–49	270 (37.7)	172 (71.1)	106 (39.3)
0–59	577 (58.0)	384 (70.8)	253 (43.8)
60–69	861 (67.0)	606 (73.5)	311 (36.1)
*P* value	< 0.0001	0.015	< 0.0001
Sex			
Male	737 (39.1)	408 (70.7)	255 (34.6)
Female	1080 (61)	729 (72.0)	476 (44.1)
P value	< 0.0001	Not Significant	< 0.0001
Education			
Primary school and less	881 (61.3)	625 (74.3)	325 (36.9)
High school	484 (46.6)	315 (70.6)	200 (41.3)
Diploma & Graduate Diploma	323 (40.6)	189 (65.2)	151 (46.7)
BSc,MSc. and Doctorate	110 (31.5)	69 (69.0)	47 (42.7)
*P* value	< 0.0001	0.024	0.016
Place of residence			
Urban (Yazd)	1601 (49.1)	1057 (70.9)	636 (39.7)
Urban (New Cities) or Semi-Urban	129 (59.2)	90 (75.6)	58 (45.0)
Rural	87 (50.3)	62 (74.7)	37 (42.5)
*P* value	0.015	Not Significant	Not Significant
Health insurance			
Yes	1733 (50.4)	1148 (71.0)	699 (40.3)
No	46 (31.1)	35 (83.3)	12 (26.1)
P value	< 0.0001	Not Significant	Not Significant
BMI (kg/m2)			
Underweight	9 (25.7)	7 (87.5)	6 (66.7)
Normal	291 (39.5)	184 (70.8)	122 (41.9)
Overweight	731 (49.6)	489 (71.7)	290 (39.7)
Obesity	766 (58.4)	515 (70.8)	310 (40.5)
P value	< 0.0001	Not Significant	Not Significant
Physical activity			
Low	1157 (55)	747 (69.9)	478 (41.3)
Moderate	576 (44.4)	410 (74.7)	221 (38.4)
High	84 (32.8)	52 (70.3)	32 (38.1)
*P* value	< 0.0001	Not Significant	Not Significant
Family history of hypertension			
Yes	1247 (80.2)	871 (72.4)	488 (39.1)
No	480 (50.1)	308 (68.6)	200 (41.7)
*P* value	< 0.0001	Not Significant	Not Significant
Family history of CVD			
Yes	686 (58.0)	472 (73.4)	319 (46.5)
No	1038 (46.5)	680 (70.6)	376 (36.2)
*P* value	< 0.0001	Not Significant	< 0.0001
Current tobacco use			
Yes	191 (34.6)	104 (61.9)	73 (38.20
No	1604 (52.4)	1095 (72.7)	647 (40.3)
*P* value	< 0.0001	0.003	Not Significant
A positive history of DM			
Yes	705 (75.2)	510 (75.3)	293 (41.6)
No	1103 (40.9)	692 (68.7)	433 (39.3)
*P* value	< 0.0001	0.002	Not Significant

^a^ Frequency in those who are aware of their hypertension

logistic regression analysis ascertained the effects of predictors of awareness, treatment, and control of hypertension. Age, sex, BMI, physical activity, insurance, and diabetes history are able to predict changes in awareness. (χ^2^ (9) = 776.154, *p* <  0.0001). It correctly classified 69.7% of cases. The logistic regression model was statistically significant for treatment (χ^2^ = 40.748, *p* = 0.001) with correct classification of 71.6% cases. Physical activity, smoking, and a history of diabetes can predict changes in treatment. The model for control of hypertension classified 62.4% of cases, correctly. Age, sex, and insurance are predictors of change to control hypertension (χ^2^ (9) = 66.724, *p* <  0.0001). Table 3 shows the contribution of each independent variable to the model and its statistical significance.

**Table 3:** Factors related to awareness, treatment, and control of hypertension in Yazd Greater Area population 20–69 years

	Aware	Treated	Controlled
	OR(95%CI)	OR(95%CI)	OR(95%CI)
Age groups			
20–29	Ref.	Ref.	Ref.
30–39	1.46 (0.90–2.39)	1.44 (0.46–4.51)	0.32 (0.12–0.87)
40–49	3.24 (2.05–5.10)	2.49 (0.85–7.26)	0.22 (0.08–0.55)
50–59	6.22 (3.97–9.75)	2.35 (0.82–6.74)	0.27 (0.11–0.67)
60–69	9.01 (5.73–14.17)	2.65 (0.92–7.59)	0.19 (0.07–0.48)
Sex			
Male	Ref.	Ref.	Ref.
Female	1.98 (1.68–2.34)	0.87 (0.67–1.11)	1.65 (1.32–2.08)
Education			
Primary school and less	Ref.	Ref.	Ref.
High school	0.98 (0.81–1.19)	0.91 (0.69–1.21)	1.23 (0.96–1.58)
Diploma and Graduate Diploma	1.22 (0.98–1.52)	0.68 (0.49–0.93)	1.66 (1.24–2.22)
BSc, MSc. and Doctorate	1.06 (0.78–1.44)	0.88 (0.54–1.44)	1.40 (0.89–2.18)
Place of residence			
Urban	1.15 (0.79–1.67)	1.16 (0.67–1.98)	1.08 (0.67–1.74)
Rural	Ref.	Ref.	Ref.
BMI (kg/m2)			
Normal	Ref.	Ref.	Ref.
Underweight	1.23 (0.52–2.94)	0.28 (0.03–2.42)	0.43 (0.09–1.86)
Overweight	1.50 (0.63–3.55)	0.27 (0.03–2.31)	0.39 (091–1.69)
Obesity	1.95 (0.82–4.62)	0.28 (0.03–2.38)	0.37 (0.08–1.59)
Physical Activity			
Low	1.17 (1.00–1.37)	0.76 (0.60–0.96)	1.19 (0.96–1.47)
Moderate/ High	Ref.	Ref.	Ref.
Insurance			
Yes	. 1.62 (1.04–2.52)	0.53 (0.23–1.24)	2.38 (1.10–5.15)
No	Ref.	Ref.	Ref.
Current tobacco use			
Yes	1.18 (0.94–1.48)	1.64 (1.13–2.37)	0.93 (0.86–1.32)
No	Ref.	Ref.	Ref.
A positive history of DM			
Yes	2.72 (2.26–3.27)	1.32 (1.05–1.67)	1.20 (0.97–1.48)
No	Ref.	Ref.	
Constant	0.035	5.56	1.75

## Discussion

The prevalence of hypertension is high among the Yazd adult population with inappropriate awareness and controlled hypertension rates. Less than half of aware patients, who were treated, had controlled blood pressure.

The results indicate that Yazd is among the areas with high blood pressure prevalence compared to similar studies in Iran and the world [12]. The prevalence of hypertension in the world has also been reported 28.8% in high and 31.5% in low-income countries [27], indicating a worsening situation in Yazd. This might be justified by the different prevalence of risk factors due to ethnicity and lifestyle changes, or different age groups in the study. Having screening intervention programs in place, may increase the difference in the prevalence of diagnosed and treated patients across regions, as well as in one area over different years.

Almost 50% of Yazdi adults are aware of their hypertension, which is lower than in high-income countries (67%) but higher than low/middle-income countries (37.9%) [27]. In different countries this awareness has been reported from 25 to 75% [28]. Awareness in Yazd is lower compared to most studies in Iran (69.2% in Isfahan, [9] 60.5% in Tabriz [12],, and 57% in Kerman [29]. It is slightly higher than Golestan’s (46.2%) and a previous study in Yazd (43.7%) [12, 16]. Over the past decade, the 5% awareness increase in Yazd was not satisfactory, considering increased access to health centers and increase in the number of health insured.

The study shows that about three-quarters of patients (71.5%), who were aware of their disease, had visited by the physician during the past three months. Although the treated hypertension in Yazd is higher than the world average (36.9%), [27] it is more inadequate than other studies in Iran [9, 12]. The availability and affordability (low cost) of health care services have made this index more favorable in Iran than in the world - even in high-income countries (55.6%) [16, 27]. Un-prescribed drug use and differences in the definition of treated people may be other reasons for this difference. Despite treatment, only 39% of the participants had controlled hypertension, which is close to the worldwide statistics (37.1%), although it was less than high-income countries (50.4%) [27]. Although the difference between treated and controlled hypertension was reported in all studies, in Iran, Isfahan (59.1%) and Tabriz (68.5%) reported a better-controlled situation [9, 12] suggesting poor control of hypertension in Yazd. In Yazd, 71.5% of those who were aware of their hypertension was visited by a physician for receiving medication. However, in both treated and untreated groups, blood pressure control did not differ (38.9% vs. 38.7%). This was lower than the result of several studies including some from developed countries, [30] and was similar to another multinational study (32.5%) [12]. Controlled hypertension was higher among females, younger age groups; health insured and educated participants which were in line with other studies [12, 31]. More physicians’ visits (by women), lack of other underlying diseases in young population, and low-cost access to health care for the insured, explain these predictors for better control of hypertension according to the regression analysis. A comparison of blood pressure control status in Yazd in this study with the previous study shows threefold growth. Since awareness and treatment of the disease have not changed, improved quality of treatment by physicians has been effective. The high awareness and uncontrolled hypertension may justify irregular follow-up by family physicians and primary health centers, especially in the urban areas.

Misuse of medication or lack of regular patient care, as well as inadequate medication administration, can be a cause of the disease poor control.

After adjustment, patients with older age, history of diabetes, female sex, and health insured were more likely to aware of their hypertension. More elderly referrals to physicians and health centers for treatment and periodic care justify older people’s awareness of their blood pressure compared to younger adults. In this study, less awareness of men than women can be due to lower access to health centers, employment, and less attention to their health. This association between age and sex has been confirmed in other studies [9, 12, 16]. However, the odds ratios between sex-age groups in geographic regions vary depending on the level of education, availability, and cost of health services.

In this study, adult education did not correlate with their awareness, which is consistent with the findings of Katibeh et al. findings in Yazd [32]. However, most studies have reported a relationship between their education and awareness of hypertension [33, 34]. Also, there was no relationship between high education and treated hypertension, as well as the control of hypertension. Higher education in people has no effect on the management of the disease, unlike the results of others, so it requires further investigation. Individuals’ health literacy appears to be more important than education for health care.

Although health insurance was not associated with patients being treated, insurance was a predictor of hypertension awareness and control. The positive effect of health insurance was also reported in other studies [35]. This can be due to the reduced cost of receiving services in continuing care.

In our study, having a history of diabetes is a predictor for awareness and treatment of hypertension. Others have confirmed that having another medical condition (diabetes, hyperlipidemia, etc.) is associated with the awareness and treatment of hypertension [9, 34, 36]. It will cause people to go to health centers; as a result, hypertension will be diagnosed sooner, and medication will be started. However, having diabetes was not a predictor for blood pressure control. Different goals in defining blood pressure control and various guidelines in diabetic patients have made blood pressure control more difficult in this group [37].

### Strengths and limitations

The strengths of this study are the large sample size with random multi-stage cluster sampling from different urban and rural areas, 95% participation rate, and, most importantly the three measurements of blood pressure at home according to the standard protocol by trained health care providers. Investigating the relationship between tobacco smoking, obesity, physical activity, and diabetes history with hypertension awareness and control are among the other advantages of this study. This study, however, had some limitations. This cross-sectional study and cannot investigate the causality. Hypertensive risk factors such as stress, dietary habits, and alcohol use have not been analyzed. Tobacco smoking, physical activity, and diabetes were self-reported which may produce bias. The details of drug adherence are an important variable for the control of hypertension, which was not recorded in this study. Also, it was not considered the relationship between economic factors and health services utilization with awareness and control of hypertension.

## Conclusion

Hypertension is a public health problem in Yazd, which is not well controlled. Half of the patients were undiagnosed, and more than half of known cases of hypertension were not controlled. It can lead to high-cost cardiovascular complications. This study represents a warning message for cardiovascular health in Yazdi adults. Health policymakers must consider new strategies for prevention. Intervention for increased screening coverage is needed, especially for men. Public awareness should be raised about hypertension and improved hypertension control under the supervision of physicians should be promoted. Implementation of family physician program for health insured persons may accelerate reaching these goals.

## Supplementary information

**Additional file 1.** YaHS questionnaire (English version)

## Data Availability

The data collected by Yazd Health Study are not open access but can be shared under conditions of collaboration and endowment. Data are available from the authors upon reasonable request and with the permission of the principal investigator. For further information, please visit YaHS website at www.yahs-ziba.com

## Abbreviations

BP: Blood Pressure
JNC: Joint National Committee
HTN: Hypertension
BMI: Body Mass Index
CI: Confidence Interval
WHO: World Health Organization
