# Supplementary material for: Awareness, treatment, and control of hypertension and related factors in adult Iranian population
Source: BMC Public Health. 2020 May 12;20:667. doi: 10.1186/s12889-020-08831-1 (PMC7216488; doi:10.1186/s12889-020-08831-1)
Supplement: Supplementary file 1 — Additional file 1. YaHS questionnaire (English version) [file 12889_2020_8831_MOESM1_ESM.pdf]

Yazd Health Study Questionnaire

Instructions:

- Please do not interview unless the informed consent signed by the interviewee

- Please do not ask the sensitive questions in front or near others

- Each question has one choice unless marked with*

- Marked questions can take up to three choices

1.Age group: 1) 20-29 2) 30-39 3) 40-49 4) 50-59 5) 60-69
2. Sex: 1) Male 2) Female
3. Length of stay in the present residential: 1) Less than 1 year 2) 2-5 years 3) 6-10 years 4) 11-15 years 5) more than 15 years
4. Are you a native of Yazd? 1) Yes >>>>> go to question 6 2) No
5. If you are not a native of Yazd, where are you from? 1) other cities of Yazd province 2) Neighboring provinces 3) Other provinces 4) Other countries
6. The respondent's education level: 1) Illiterate 2) Junior high school 3) High school diploma or associates degree 4) Bachelor's degree 5) Masters degree or higher
7. The number of household members (including the interviewee): 1) 2 2) 3 3) 4 4) 5 5) 6 and more
8. Marital status: 1) Married 2) Single 3) Widowed 4) Divorced
9. Systolic blood pressure (mm Hg) (based on measurements): 1) Below 90 2) 90-119 3) 120-139 4) 140-159 5) 160 and above
10. Diastolic blood pressure (mm Hg) (based on measurements): 1) Below 70 2) 70-79 3) 80-89 4) 90-99 5) 100 and above
11. Range of body mass index (based on measurements): 1) Less than 18.5 2) 18.5-24.9 3) 25-29.9 4) 30-39.9 5) 40 and above
A) Physical activity questions
12. How often do you do vigorous physical activity, such as running or heavy physical work (where your heart rate rises or you sweat)? 1) Never 2) Once a week 3) Twice a week 4) Three to four times a week 5) Five or more times a week
13. How long does each of your vigorous physical activity sessions last (minutes) ? 1) Less than 10 minutes 2) 10-20 minutes 3) 20-30 minutes 4) 30-60 minutes 5) More than 60 minutes
14. How often do you do moderate physical activity, such as moderate activities and fast walking (where you sweat a little or do not get tired)? 1) Never 2) Once a week 3) Twice a week 4) Three or four times a week 5) Five or more times a week
15. How long does each of your moderate physical activity sessions last (minutes) ? 1) Less than 10 minutes 2) 10-20 minutes 3) 20-30 minutes 4) 30-60 minutes 5) More than 60 minutes
16. How often do you do low physical activities, such as slow walking or sedentary works (where you make little effort or do not sweat)? 1) Never 2) Once a week 3) Twice a week 4) Three or four times a week 5) Five or more times a week
17. How long does each of your low physical activity sessions last (minutes) ? 1) Less than 10 minutes 2) 10-20 minutes 3) 20-30 minutes 4) 30-60 minutes 5) More than 60 minutes

B - Sleep status questions

18. In the past month, what time did you usually go to bed at night? 1) 8-9 pm 2) 10-11 pm 3) 12 pm -1 am 4) 2-3 am 5) After 3 am
19. In the past month, what time did you usually wake up in the morning? 1) 4-5 am 2) 6-7 am 3) 8-9 am 4) 10-11 am 5) After 11 am
20. In the past month, how long did you actually sleep through the day? 1) Less than 5 hours 2) 6-7 hours 3) 8-10 hours 4) More than 10 hours
21. In the past month, how long did it usually take you to sleep at night? 1) Immediately 2) Less than 15 minutes 3) 15-30 minutes 4) 30-60 minutes 5) More than 60 minutes
22. In the past month, how often did you use sleeping pills or tranquilizers to sleep?<br>1) I had no experience 2) Less than once a week 3) Once or twice a week 4) Three or more times a week
23. In the past month, how often could you not fall asleep within 30 minutes? 1) I had no experience 2) Less than once a week 3) Once or twice a week 4) Three or more times a week
24. In the past month, how often did you wake up at midnight to go to toilet? 1) I had no experience 2) Less than once a week 3) Once or twice a week 4) Three or more times a week
25. In the past month, how often did you wake up at night or early in the morning? 1) I had no experience 2) Less than once a week 3) Once or twice a week 4) Three or more times a week
26. In the past month, how often did you have nightmares? 1) I had no experience 2) Less than once a week 3) Once or twice a week 4) Three or more times a week
27. How many hours a day do you usually watch TV?<br>1) Less than 1 hour 2) 1-3 hours 3) 4-6 hours 4) More than 6 hours 5) Not at all
28. How many hours a day do you usually watch satellite, television, or movies?<br>1) Less than 1 hour 2) 1-3 hours 3) 4-6 hours 4) More than 6 hours 5) Not at all

C - Mental health

29. In the past week, how much time did you spend with your family and friends?<br>1) Not at all 2) 1-3 hours 3) 4-7 hours 4) 8-12 5) More than 12 hours
30. In the past week, how long did you talk with your family and friends on the phone or mobile phone? 1) Not at all 2) 1-3 hours 3) 4-7 hours 4) 8-12 5) More than 12 hours
31 In the past week, how long did you spend in religious and social groups?<br>1) Not at all 2) 1-3 hours 3) 4-7 hours 4) 8-12 5) More than 12 hours
32. How many close friends (to whom you could say your secrets), except for your family members, do you have? 1) 1-2 2) 3-4 3) 5-6 4) More than 6 7) Not at all

Mental status questions:

Some sentences that people have used to introduce you have been gathered below.
Read all the sentences to the respondents and according to the following scale, write the number that indicates how the person feels at the moment. There are no right or wrong answers.

Do not spend a lot of time for each sentence. Submit a response that seems to best describe how the person feels at the moment.

Sentences				
33. It is difficult for me to stay calm.	1) Not at all	2) Somewhat	3 moderately)	4) too much
34. I noticed that my mouth is dry.	1) Not at all	2) Somewhat	3) moderately	4) too much
35. I do not think I can feel good experience.	1) Not at all	2) Somewhat	3 moderately	4) too much
36. I had trouble breathing.	1) Not at all	2) Somewhat	3 moderately	4) too much
37. It is difficult to volunteer for work.	1) Not at all	2) Somewhat	3 moderately	4) too much
38. In my situation, I reacted wildly.	1) Not at all	2) Somewhat	3 moderately	4) too much
39. I feel the tremor in my body.	1) Not at all	2) Somewhat	3 moderately	4) too much
40. I feel I use a lot of mental energy.	1) Not at all	2) Somewhat	3 moderately	4) too much
41. I am worried about my panic or stupid actions in some situations.	1) Not at all	2) Somewhat	3 moderately	4) too much
42. I feel I do not have something that I have been waiting for it.	1) Not at all	2) Somewhat	3 moderately	4) too much
43. I feel I am distressed and quandary.	1) Not at all	2) Somewhat	3 moderately	4) too much
44. Calmness and serenity is difficult for me.	1) Not at all	2) Somewhat	3 moderately	4) too much
45. I feel heart dead and heart broken.	1) Not at all	2) Somewhat	3 moderately	4) too much
46. I do not keep patience in confront to anything that limit my work.	1) Not at all	2) Somewhat	3 moderately	4) too much
47. I feel that I may fear at any moment.	1) Not at all	2) Somewhat	3 moderately	4) too much
48. I am not able to show my enthusiasm about many things.	1) Not at all	2) Somewhat	3 moderately	4) too much
49. I feel I am not highly valuable as an individual.	1) Not at all	2) Somewhat	3 moderately	4) too much
50. I think I am very irritable and sensitive.	1) Not at all	2) Somewhat	3 moderately	4) too much
51. Without any physical activity, I notice that my heart works unusually.	1) Not at all	2) Somewhat	3 moderately	4) too much

(e.g., rapid heartbeat or I feel heart failure for some moments).				
52. I fear with no acceptable reasons.	1) Not at all	2) Somewhat	3 moderately	4) too much
53. I feel that life is meaningless.	1) Not at all	2) Somewhat	3 moderately	4) too much

D - Questions about quality of life:

54. How do you assess your health status in the past month? 1) Very good 2) Good 3) Intermediate 4) Bad 5) Very bad
55. In the past month, how much have your physical activities (such as climbing stairs or walking) been limited due to physical health problems? 1) Not at all 2) Very low 3) Somewhat 4) A lot 5) I could not move
56. In the past month, how much have your daily works inside and outside the house been limited due to physical health problems? 1) Not at all 2) Very low 3) Somewhat 4) A lot 5) I could not move
57. How much bodily pain have you had during the past month? 1) Not at all 2) Very low 3) Somewhat 4) A lot 5) too much
58. How much bodily energy did you have during the past month? 1) too much 2) A lot 3) Somewhat 4) Low 5) Not at all
59. Over the past month, how much physical and mental problems have restricted your social relationships with your family and friends? 1) Not at all 2) Very low 3) Somewhat 4) A lot 5) I could not have relationship
60. Over the past month, how much have mental problems, such as anxiety and depression, hurt you? 1) Not at all 2) Very low 3) Somewhat 4) A lot 5) too much
61. Over the past month, how much have physical and mental problems restricted your daily activities? 1) Not at all 2) Very low 3) Somewhat 4) A lot 5) I could not do my daily activities

E- Questions about chronic diseases:

Which of the following diseases has been diagnosed by your doctor?

62. Heart disease 1) Yes 2) No >>>>> Go to question 70
63. If yes, what type? 1) Valvular heart disease 2) Heart failure 3) Irregular heartbeat (arrhythmia) 4) Intra-aortic angioblasty 5) Open-heart surgery
64. In case of having any of the above diseases, how many years ago did it start? 1) Less than 1 year 2) 1-2 years 3) 3-4 years 4) 5-6 years 5) 7 or more years ago
65. When was the last time you went to a doctor for your disease? 1) Less than 1 month ago 2) 1-3 months ago 3) 4-5 months ago 4) 6-12 months ago 5) More than 1 year ago
66. Have you ever felt pain, discomfort, or pressure and heaviness in your chest or under the breast bone? 1) Yes 2) No
67. Do you have pain in your chest when you walk slowly or quickly or go up the hill? 1) Yes 2) No
68. Does your pain goes away when you change your

situation (standing, sitting, or walking slower) or use sublingual tabs? 1) Yes 2) No
69. Is the pain eliminated in less than 10 minutes? 1) Yes 2) No
70. Do your first degree relatives (father, mother, sister, brother, children) have a history of heart disease? 1) Yes 2) No
71. High blood pressure (hypertension)? 1) Yes 2) No >>> Go to question 77
72. If you have the disease, how many years ago did it start? 1) Less than one year ago 2) 1-2 years ago 3) 3-4 years ago 4) 5-6 years ago 5) 7 or more years ago
73. When was the last time you went to a doctor for your disease? 1) Less than a month ago 2)1-3 months ago 3) 4 to 6 months ago 4) 6 months to a year ago 5) Over a year ago
74. Do your first degree relatives (father, mother, sister, brother, children) have a history of high blood pressure (hypertension)? 1. Yes 2. No
75. Do you have a history of mental illness? 1) Yes 2) No
76. If yes, what type? 1) Cerebral hemorrhage 2) Strokes 3) Seizure 4) Brain Cancer 5) Other
77. Do your first degree relatives (father, mother, sister, brother, children) have a history of mental illness? 1) Yes 2) No
78. Do you have nocturnal snoring problem? 1) Yes 2) No
79. Do you have a family history of sudden death? 1) Yes 2) No
80. Diabetes: 1) Yes 2) No >> Go to question 88
81. If you have the disease, how many years ago did it start? 1) Less than one year ago 2) 1-2 years ago 3) 3-4 years ago 4) 5-6 years ago 5) 7 or more years ago
82. Which method do you use to control diabetes? 1) Dietary 2) Herbal extract 3) Tablets 4) Insulin 5) None
83. Do you take medicine for diabetes regularly? 1) Yes 2) No
84. If you use insulin, how many years after the diagnosis did you start taking it? 1) Immediately 2) 6 months to a year 3) 1 to 3 years 4) 3 to 10 years 5) More than ten years
85. When was the last time you went to a doctor for your disease? 1) 3 to 6 months ago 2) 7 months to one year ago 3) 2 to 3 years ago 4) 4 to 10 years ago 5) More than ten years ago
86. What type of physician did you refer to? 1) General 2) Additional domestic specialists 3) Endocrinologist
87. Which of diabetes complications appeared to you at first? 1) Gestational diabetes 2) Ocular complications of diabetes 3) Renal complications of diabetes 4) Diabetic foot 5) Wounds caused by diabetes
88. Do your first degree relatives (father, mother, sister, brother, children) have a history of this disease? 1) Yes 2) No
89. Increased blood cholesterol 1) Yes 2) No >>> Go to question 92
90. If you have the disease, how many years ago did it start? 1) Less than one year ago 2) 1-2 years ago 3) 3-4 years ago 4) 5-6 years ago 5) 7 or more years ago
91. When was the last time you went to a doctor for your disease? 1) Less than a month ago 2) 2 to 3 months ago 3) 4 to 6 months ago 4) 7 months to one year ago 5) More than a year ago
92. Do your first degree relatives (father, mother, sister, brother, children) have a history of this disease? 1) Yes 2) No
93. Blood coagulation problems 1. Yes 2. No >>> Go to question 97

94. If you have the disease, how many years ago did it start? 1) Less than one year ago 2) 1-2 years ago 3) 3-4 years ago 4) 5-6 years ago 5) 7 or more years ago
95. When was the last time you went to a doctor for your disease? 1) Less than a month ago 2) 2 to 3 months ago 3) 4 to 6 months ago 4) 7 months to one year ago 5) More than a year ago
96. Do you take medicine to control the clotting regularly? 1) Yes 2) No
97. Do your first degree relatives (father, mother, sister, brother, children) have a history of this disease? 1) Yes 2) No
98. Asthma 1) Yes 2) No >>> Go to question 101
99. If you have the disease, how many years ago did it start? 1) Less than one year ago 2) 1-2 years ago 3) 3-4 years ago 4) 5-6 years ago 5) 7 or more years ago
100. When was the last time you went to a doctor for your disease? 1) Less than a month ago 2) 2 to 3 months ago 3) 4 to 6 months ago 4) 7 months to one year ago 5) More than a year ago
101. Do your first degree relatives (father, mother, sister, brother, children) have a history of this disease? 1) Yes 2) No
102. Thyroid problems 1) Yes 2) No>>> Go to question 106
103. What type of thyroid problems do you have? 1) Hypothyroidism 2) Hyperthyroidism 3) Goiter 4) Malignant (cancerous)
104. If you have the disease, how many years ago did it start? 1) Less than one year ago 2) 1-2 years ago 3) 3-4 years ago 4) 5-6 years ago 5) 7 or more years ago
105. When was the last time you went to a doctor for your disease? 1) Less than a month ago 2) 2 to 3 months ago 3) 4 to 6 months ago 4) 7 months to one year ago 5) More than a year ago
106. Do your first degree relatives (father, mother, sister, brother, children) have a history of this disease? 1) Yes 2) No
107. Depression 1) Yes 2) No >>> Go to question 110
108. If you have the disease, how many years ago did it start? 1) Less than one year ago 2) 1-2 years ago 3) 3-4 years ago 4) 5-6 years ago 5) 7 or more years ago
109. When was the last time you went to a doctor for your disease? 1) Less than a month ago 2) 2 to 3 months ago 3) 4 to 6 months ago 4) 7 months to one year ago 5) More than a year ago
110. Do your first degree relatives (father, mother, sister, brother, children) have a history of this disease? 1) Yes 2) No
111. Alzheimer's disease 1) Yes 2) No >>> Go to question 114
112. If you have the disease, how many years ago did it start? 1) Less than one year ago 2) 1-2 years ago 3) 3-4 years ago 4) 5-6 years ago 5) 7 or more years ago
113. When was the last time you went to a doctor for your disease? 1) Less than a month ago 2) 2 to 3 months ago 3) 4 to 6 months ago 4) 7 months to one year ago 5) More than a year ago
114. Do your first degree relatives (father, mother, sister, brother, children) have a history of this disease? 1) Yes 2) No
115. Osteoporosis 1) Yes 2) No >>> Go to question 120
116. If you have the disease, how many years ago did it start? 1) Less than one year ago 2) 1-2 years ago 3) 3-4 years ago 4) 5-6 years ago 5) 7 or more years ago
117. Did you have a bone fracture after diagnosis of your osteoporosis? 1) Yes 2) No

118. When was the last time you went to a doctor for your disease? 1) Less than a month ago 2) 2 to 3 months ago 3) 4 to 6 months ago 4) 7 months to one year ago 5) More than a year ago
119. Is family history of osteoporosis associated with fractures? 1) Yes 2) No
120. Do your first degree relatives (father, mother, sister, brother, children) have a history of this disease? 1) Yes 2)No
121. Joint pain (arthritis) 1) Yes 2) No >>> Go to question 125
122. If you have the disease, which of your joints are affected: 1) Neck 2) Back 3) Waist 4) Knee 5) Small joints of the foot
123. If you have the disease, how many years ago did it start? 1) Less than one year ago 2) 1-2 years ago 3) 3-4 years ago 4) 5-6 years ago 5) 7 or more years ago
124. When was the last time you went to a doctor for your disease? 1) Less than a month ago 2) 2 to 3 months ago 3) 4 to 6 months ago 4) 7 months to one year ago 5) More than a year ago
125. Do your first degree relatives (father, mother, sister, brother, children) have a history of this disease? 1) Yes 2) No
126. Skin cancer 1) Yes 2) No >> Go to question 129.
127. If you have the disease, how many years ago did it start? 1) Less than one year ago 2) 1-2 years ago 3) 3-4 years ago 4) 5-6 years ago 5) 7 or more years ago
128. When was the last time you went to a doctor for your disease? 1) Less than a month ago 2) 2 to 3 months ago 3) 4 to 6 months ago 4) 7 months to one year ago 5) More than a year ago
129. Do your first degree relatives (father, mother, sister, brother, children) have a history of this disease? 1) Yes 2) No
130. Breast cancer (ask women only) 1. Yes 2. No >> Go to question 133
131. If you have the disease, how many years ago did it start? 1) Less than one year ago 2) 1-2 years ago 3) 3-4 years ago 4) 5-6 years ago 5) 7 or more years ago
132. When was the last time you went to a doctor for your disease? 1) Less than a month ago 2) 2 to 3 months ago 3) 4 to 6 months ago 4) 7 months to one year ago 5) More than a year ago
133. Do your first degree relatives (father, mother, sister, brother, children) have a history of this disease? 1) Yes 2) No
134. Lung cancer 1) Yes 2) No >>> Go to question 137
135. If you have the disease, how many years ago did it start? 1) Less than one year ago 2) 1-2 years ago 3) 3-4 years ago 4) 5-6 years ago 5) 7 or more years ago
136. When was the last time you went to a doctor for your disease? 1) Less than a month ago 2) 2 to 3 months ago 3) 4 to 6 months ago 4) 7 months to one year ago 5) More than a year ago
137. Do your first degree relatives (father, mother, sister, brother, children) have a history of this disease? 1) Yes 2) No
138. Colon cancer 1. Yes 2. No >>> Go to question 141
139. If you have the disease, how many years ago did it start? 1) Less than one year 2) 1-2 years ago 3) 3-4 years ago 4) 5-6 years ago 5) 7 or more years ago
140. When was the last time you went to a doctor for your disease? 1) Less than a month ago 2) 2 to 3 months ago 3) 4 to 6 months ago 4) 7 months to one year ago 5) More than a year ago

141. Do your first degree relatives (father, mother, sister, brother, children) have a history of this disease? 1) Yes 2) No
142. Stomach cancer 1. Yes 2. No >> Go to question 145
143. If you have the disease, how many years ago did it start? 1) Less than one year ago 2) 1-2 years ago 3) 3-4 years ago 4) 5-6 years ago 5) 7 or more years ago
144. When was the last time you went to a doctor for your disease?1) Less than a month ago 2) 2 to 3 months ago 3) 4 to 6 months ago 4) 7 months to one year ago 5) More than a year ago
145 Do your first degree relatives (father, mother, sister, brother, children) have a history of this disease? 1) Yes 2) No
146. Colorectal cancer 1. Yes 2. No >>> Go to question 149
147. If you have the disease, how many years ago did it start? 1) Less than one year ago 2) 1-2 years ago 3) 3-4 years ago 4) 5-6 years ago 5) 7 or more years ago
148. When was the last time you went to a doctor for your disease? 1) Less than a month ago 2) 2 to 3 months ago 3) 4 to 6 months ago 4) 7 months to one year ago 5) More than a year ago
149. Do your first degree relatives (father, mother, sister, brother, children) have a history of this disease? 1) Yes 2) No

150. Prostate cancer (Ask men only) 1. Yes 2. No >> Go to question 153
151. If you have the disease, how many years ago did it start? 1) Less than one year ago 2) 1-2 years ago 3) 3-4 years ago 4) 5-6 years ago 5) 7 or more years ago
152. When was the last time you went to a doctor for your disease? 1) Less than a month ago 2) 2 to 3 months ago 3) 4 to 6 months ago 4) 7 months to one year ago 5) More than a year ago
153. Do your first degree relatives (father, mother, sister, brother, children) have a history of this disease? 1) Yes 2) No
154. Ovarian cancer (Ask women only) 1. Yes 2. No >> Go to question 157
155. If you have the disease, how many years ago did it start? 1) Less than one year ago 2) 1-2 years ago 3) 3-4 years ago 4) 5-6 years ago 5) 7 or more years ago
156. When was the last time you went to a doctor for your disease? 1) Less than a month ago 2) 2 to 3 months ago 3) 4 to 6 months ago 4) 7 months to one year ago 5) More than a year ago
157. Do your first degree relatives (father, mother, sister, brother, children) have a history of this disease? 1) Yes 2) No
**(F) surgery questions:**
158. Have you had a bladder surgery? 1. Yes 2. No >> Go to question 160
159. Your age at the time of surgery: 1) Below 30 years 2) 30-39 years 3) 40-49 years 4) 50-59 years 5) 60 years and above
160. Have you had a kidney stone surgery? 1) Yes 2) No >> Go to question 162
161. Your age at the time of surgery: 1) Below 30 years 2) 30-39 years 3) 40-49 years 4) 50-59 years 5) 60 years and above
162. Have you had a knee replacement surgery? 1) Yes 2) No >> Go to question 164
163. Your age at the time of surgery: 1) Below 30 years 2) 30-39 years 3) 40-49 years 4) 50-59 years 5) 60 years and above
164. Have you had a hip surgery? 1. Yes 2. No >> Go to question 166

165. Your age at the time of surgery: 1) Below 30 years 2) 30-39 years 3) 40-49 years 4) 50-59 years 5) 60 years and above
166. Have you had a gallbladder surgery? 1. Yes 2. No >> Go to question 168
167. Your age at the time of surgery: 1) Below 30 years 2) 30-39 years 3) 40-49 years 4) 50-59 years 5) 60 years and above
168. Have you had an open heart surgery? 1. Yes 2. No >> Go to question 170
169. Your age at the time of surgery: 1) Below 30 years 2) 30-39 years 3) 40-49 years 4) 50-59 years 5) 60 years and above
170. During the past month, did you have pain in the joints of your neck? 1) Yes 2) No
171. During the past month, did you have pain in the joints of your back? 1) Yes 2) No
172. During the past month, did you have pain in your knee joints? 1) Yes 2) No
173. During the past month, did you have a history of gout (pain in the small joints of the foot)? 1) Yes 2) No
174. Total number of days in your life that you have been hospitalized? 1) Never 2) 1-10 days 3) 11-20 days 4) 21-30 days 5) More than one month
175. If you were hospitalized in the first 6 months of 2013, how was your satisfaction level with the health services? 1) Very good 2) Good 3) Moderate 4) Bad 5) Very bad
176. If you were admitted in hospital on 5 May 2014, how was your satisfaction level with the health services? 1) Very good 2) Good 3) Moderate 4) Bad 5) Very bad
177. Over the past year, how many times have you gone to a doctor? 1) Never 2) 1 3) 2 4) 3-4 5) 6 and more
178. Over the past year, how many times have you gone to a specialist? 1) Never 2) 1 3) 2 4) 3-4 5) 6 and more
179. If you get sick, where will you refer for the first time? 1) Health centers (clinics) 2) General practitioner 3) Specialist 4) Fellowship 5) Traditional practitioner
**G. Oral and dental questions:**
180. How many times a day do you use toothbrush? 1) Never 2) Once a day 3) Twice a day 4) Three times a day 5) Some times
181. Do you use dental floss? 1. Yes 2. No 3.) Occasionally
182. Do you have full dentures? 1. Yes 2. No
183. How many of your teeth are artificial? 1) None 2) 1-2 3) 3-4 4) 5-6 5) 7 and more
184. Number of drawn teeth? 1) None 2) 1-3 3) 4-6 4) 7-8 5) 9 and more
185. The number of filled teeth (approximately)? 1) None 2) 1-3 3) 4-6 4) 7-8 5) 9 and more
186. How often do you go to a dentist on average? 1) Never 2) In case of pain 3) at regularly examination period 4) Once a year 5) 2-3 times a year
**H- Accidents**
**In the past year, which of the following events happened to you?**
187. 1) Traffic accident 2) Fall 3) Drowning 4) Scoot
188. 1) Poisoning 2) Burn 3) Animal bites 4) Attempt for suicide 5) Fall into a pit
189. 1) Electric shock 2) Work-related incident 3) Bone fractures 4) Dealing with sharp objects 5) None>> Go to question 195

**Your status in traffic accident:**
190.1) Motorcyclist 2) Pedestrian 3) Driver of cargo or private cars 4) Passenger of private cars 5) Passenger of cargo veicles
191. 1) Bus driver 2) Minibus driver 3) Lorry driver 4) Passenger of bus or minibus 5) Cyclist
**The front vehicle in the accident was:**
192. 1) Private car 2) Goods van 3) Motorcycle 4) Bus 5) Minibus
193. 1) Truck 2) Bicycle 3) Fixed objects 4) Other
194. The result of the accident 1) Recovered without referring to medical centers 2) Discharged after examination 3) Under treatment 4) Recovered 5) Disabled
195. How do you go to work**?** .1) Personal car 2) Motorcycle 3) Bicycle 4) Walking 5) Public transportation
196. Do you fasten your seat belt when using a private or public vehicle? 1) Only in my personal car 2. Only in public vehicles 3. Both 4. None
**I - FAQ habits**
197. What do you often drink with food? 1) Mineral water 2) Carbonated drinks 3) Juice 4) Yogurt 5) Tap water
198. How often do you drink carbonated beverages? 1) Never 2) Less than once a week 3)2-1 times a week 4) 3-4 times a week 5) 5 or more times a week
199. How often do you use sweetened drinks with sugar, such as homemade syrup and packaging juice? 1) Never 2) Less than once a week 3) Once a week 4) 2-3 times a week 5) 4 or more times a week
200. What is the main reason for you to use fast food? (Mark only one answer from the 10 options) 1) Children’s tendency 2) Excessive work 3) Flavorful and good taste 4) rapid cooking 5) Cheapness and availability
201. 1) Advertising 2) As a hobby 3) Possibility of being with family and friends 4) Other 5) I do not eat fast food
202. On average, how much do you spend on fast food every month? 1) None 2) Less than 50 thousand Tomans 3) 50-100 thousand Tomans 4) 101-150 thousand Tomans 5) More than 150 thousand Tomans or you can write $ equivalent
**How often do you use the food categories listed below?**
203. Hamburgers, hot dogs, sausages, pizza: 1) Once a day 2) 1-3 times a week 3) 1-3 times a month 4) 4-10 times a year 5) Never
204. French fries, Samosa, falafel, nugget: 1) Once a day 2) 1-3 times a week 3) 1-3 times a month 4) 4-10 times a year 5) Never
205. Olivier salad: 1) Once a day 2) 1-3 times a week 3) 1-3 times a month 4) 4-10 times a year 5) Never
206. Canned food: : 1) Once a day 2) 1-3 times a week 3) 1-3 times a month 4) 4-10 times a year 5) Never
207. How often do you eat breakfast: 1) Never 2) Once a week 3) 2-3 times a week 4) 4-5 times a week 5) Every day
**Typically, how many times a week you use the following foods:**
208. Egg: 1) Never 2) Once 3) 2-3 times 4) 4-5 times 5) Every day
209. Meat: 1) Never 2) Once 3) 2-3 times 4) 4-5 times 5) Every day
210. Chicken: 1) Never 2) Once 3) 2-3 times 4) 4-5 times 5) Every day
211. Beans: 1) Never 2) Once 3) 2-3 times 4) 4-5 times 5) Every day
212. How do you usually cook fish? 1) Frying 2) boiling / steaming 3) Barbecuing

4) Baking in oven 5) Other
213. How many times a day do you eat candies (size of a sugar cube and more)? 1) Not at all 2) Once 3) 2-3 times 4) 4-6 times 5) 7 and more times
214. How many times a day do you eat middle-sized sugar cubes? 1) Not at all 2) 1-2 times 3) 3-4 times 4) 5-8 times 5) 9 and more times
215. How many times a week do you use fried food? 1) Not at all 2) Once 3) 2-3 times 4) 3-4 times 5) 5 and more times
216. What kind of oil do you usually use for cooking at home? 1) Vegetable oil 2) Animal fat or butter<br>3) Animal oil 4) Olive oil, sesame oil 5) other liquid oils
217. What method of cooking do you use most? 1) Boiling 2) Frying 3) Barbecuing<br>4) Cooking in the oven 5) Steaming
218. How many times a week do you eat cakes, muffins, and biscuits? 1) Not at all 2) Once 3) 2-3 times 4) 3-4 times 5) 5 and more times
219. How many times a week do you eat snacks, such as potato chips and crunchy? 1) Not at all 2) Once 3) 2-3 times 4) 3-4 times 5) 5 and more times
220. If each unit of vegetables equals to half a cup of cooked vegetables or one cup of raw vegetables or salad, how many units of vegetables do you averagely consume per day? 1) Not at all 2) One unit 3) 2-3 units 4) 4-5 units 5) 6 and more units

222. If each unit of fruit equals to a medium size fruit, two small size fruits, one cup of chopped fruit, or half a glass of fruit juice, how many units of fruit do you consume a day?<br>1) Not at all 2) One unit 3) 2-3 units 4) 4-5 units 5) 6 and more units
223. How much fat do you remove from meat before eating or cooking meat foods?<br>1) All visible fat<br>2) Most visible fat 3) Little visible fat 4) I do not remove fat 5) I do not eat meat
224. Do you remove chickens' skin before cooking or eating it? 1) Never 2) Occasionally 3) Sometimes 4) Usually 5) Always
225. How many glasses of water do you drink every day? 1) None 2) 1-3 glasses 3) 4-6 glasses 4) 7-8 glasses 5) 9 and more glasses
226. How many glasses of milk do you drink every week? 1) None 2) 1-2 3) 3-4 4) 5-6 5) 7 and more
227. How many cups of yogurt you eat every week? 1) None 2) 1-2 3) 3-4 4) 5-6 5) 7 and more
228. How many times a week do you eat cheese? 1) Not at all 2) Once 3) 2 times<br>4) 3 times 5) 4 and more times
229. Over the past year, what kind of dairy products have you consumed? 1) Uncontrolled fat dairy products 2) Dairy products with averagely 3% fat 3) Low fat, 1.5 to 2% fat 4) Do not know 5) I do not use dairy products
230. Which of the following medications or supplements have you used during the past month?<br>1) Iron supplement 2) Calcium supplement 3) Vitamin supplement 4) Other supplements 5) None >> Go to question 23
231. Were the above medications prescribed by a doctor? 1) Yes 2) No
232. Do you put salt on the table? 1) Yes 2) No
233. Do you use module spoonto add salt in cooking? 1) Yes 2) No 3) I do not know
J. Occupation

234. are you employed: 1) Yes 2) Housewife 3) No>> Go to question 237
235. If the answer is "yes", how many hours a day do you work? 1) Less than one hour 2) 1 to 3 hours 3) 4 to 6 hours 4) 7 to 9 hours 5) More than 9 hours
236. Your occupation type: 1) Governmental 2) Private 3)Technical Jobs 4) Service jobs 5) Unpaid jobs
237. What kind of insurance do you use:<br>1) I do not have insurance 2) Medical services insurance 3) Social security insurance 4) Iranian national insurance 5) Others
238. How many hours do you usually sit at home every day? 1) Less than one hour 2) 1-3 hours 3) 4-6 hours 4) 7-9 hours 5) 10 hours or more
239. How many hours do you usually sit down at work or in public or private motor vehicles every day? 1) Less than one hour 2) 1 to 3 hours 3) 4 to 6 hours 4) 7-9 hours 5) More than 9 hours
240. How many hours a day do you spend on the Internet? 1) Never 2) Less than 30 minutes 3) 30-60 minutes 4) 1-3 hours 5) 4 hours and more
241. How many hours a day do you spend on talking with others on using (Viber, Tango, WhatsApp)? 1) Never 2) Less than 30 minutes 3) 30-60 minutes 4) 1-3 hours 5) 4 hours and more
K- Traditional medicine questions
242. Have you ever referred to a traditional practitioner? 1) Yes 2) No >>> Go to question 254
243. Which of the following therapies of traditional medicine have you used?<br>1) Herbal tradition 2) Traditional medicine diet 3) Massage therapy 4) Blood-letting 5) Others
244. 1) Cupping 2) Hirudo therapy 3)FASD 4) Hydrotherapy
245. To improve which of the following diseases did you use the above methods? (Only one answer) 1) Diabetes 2) Hypertension 3) High fat 4) Fatty liver 5) Headache (migraine)
246. 1) Joint pain 2) Obesity 3) Women’s disorders 4) Skin disorders 5) Psychiatric disorders
247. How many times did you use this method? 1) Once 2) Twice 3) 3 times 4) Four times 5) Five and more times
248. Who recommended using traditional medicine to you? 1) Friends and family 2) Apothecary (Attar) 3) Traditional practitioner (Hakim) 4) GP 5) Specialist
249. Does your healthcare provider (modern medicine) know about your traditional medicine treatment? 1) Yes 2) No
250. If your doctor does not know about your traditional medicine treatment, what is the reason?<br>1) Fear from the conventional medicine physician’s inappropriate behavior<br>2) Fear from the conventional medicine physician’s discomfort<br>3) Fear from the conventional medicine physician’s inattention because of reference to the traditional medicine specialist 4) Fear from being rejected by the conventional medicine doctor<br>5) Fear from discontinuation of treatment by the conventional medicine doctor
251. Are you aware of the effects of traditional medicine? 1) Yes 2) No
252. In the past year, which medicinal plants have you used? 1) Thyme, chamomile flowers, properties 2) Sisymbrium irio, Borage, 3) Lavender 4) Valerian

**L - Smoking and drugs:**
253. Do you smoke?<br>1. Yes 2. Sometimes 3. I have quitted smoking 4. No (use less than 100 cigarettes) >>> Go to question 258
254. How many members of your family, other than you, smoke cigarettes? 1. None 2. 1 3. 2 4. 3 and more
255. How many years have you smoked cigarettes? 1) Less than one year 2) 1-5 years 3) 6-10 years 4) 11 and more years
256. How many cigarettes do you smoke daily? 1) 1-10 2) 11-20 3) 21-40 4) More than 41
257. How many years ago did you quit smoking? 1) Less than one year 2) 1-5 years 3) 6-9 years 4) 10 or more years
258. Do you use hookah? 1) Yes 2) No >>>> Go to question 260 3) Occasionally
259. If yes, how many times a week? 1) Once 2) 2-3 times 3) 4-7 times 4) More than once a day
260. Do you use drugs? 1) No >>>> Go to question 263. 2) occasionally 3) Yes
261. If yes, how many times per week? 1) Once 2) 2-3 times 3) 4-7 times 4) More than once a day
262. Do you have a history of addiction treatment? 1. Yes 2. No
263. Do you have a family history of addiction treatment? 1. Yes 2. No
**M - other questions:**
264. Home ownership: 1) Personal 2. Rental 3- Organizational 4. Other
265. House infrastructure area: 1) Under 50 m^2. 2) 50-100 m^2 3) 101-200 m^2 4) 201 m^2 and more
266. How often do you read books (except for the Bible and prayer books) in a day? (1) Not at all 2) Less than 30 minutes 3) 30- 60 min 4) More than an hour
267. Do you believe in magic? 1) Yes 2) No
268. Do you believe in evil eye? 1) Yes 2) No
269. Over the past year, how many times have you traveled outside the province? 1) Never 2) 1-3 times 3) 4-8 times 4) 9 times or more
270. How many times have you traveled overseas? 1) Never 2) 1-3 times 3) 4-8 times 4) 9 times or more
271. Among these alternatives, what do you consider as the most important social damage to Yazd? 1) Backbiting 2) Telling lies 3) Lawlessness 4) Not following Sharia laws/regulations
272. Are you satisfied with your family? 1) Yes 2) No
273. Are you Zoroastrian? 1) Yes 2) No
**Here men's interview is finished**<br>>>>
**N – Women's special questions**
274. Are you married? 1. Yes 2. No >> The interview is finished.
275. How many years have you been married? 1) Less than 2 years 2) 3-5 years 3) 6-9 years 4) 10 years and more
276. How many vaginal deliveries have you had? 1) None 2) 1 3) 2 4) 3 and more
277. Considering the increase in our aging population and small number of children in Iranian families, do you want more children? 1) Yes 2) I do not think so 3) No
278. Your age at the first delivery. 1) Less than 15 years 2) 16-20 years

3) 21-34 years 4) 35 years and above
279. Your age at the last delivery. 1) Less than 15 years 2) 16-20 years 3) 21-34 years 4) 35 years and above
280. Did you have a miscarriage or stillbirth? 1. Yes 2. No
281. If yes, how many? 1) 1 2) 2 3) 3 4) 4 and more
282. Are you currently pregnant? 1) Yes 2) No 3) I am not sure
283. Did you present with any of the following side effects in your previous pregnancy? 1) Premature delivery 2) Gestational hypertension 3) Gestational diabetes 4) Placental delivery
284. If you had postpartum hemorrhage, how were you treated? 1) Removing the uterus 2) Drug therapy
285. Which one of the following screening tests have you done? 1) Pap smear 2) Mammography 3) Ultrasound (other than pregnancy ultrasound) 4) None
286. Do you have a family history of infertility (sister, mother, daughter, aunt, daughter, uncle, etc.)? 1. Yes 2. No
287. Have you had infertility problems (one year absence of pregnancy without contraceptive methods)? 1. Yes 2. No
288. Is your infertility primary (you do not get pregnant) or secondary (pregnancy will not be delivered)? 1) Primary 2) Secondary
289. What is your infertility problem? 1) Male 2) Female 3) Both 4) None
290. How long did you breastfeed your children? 1) I did not breastfeed my children 2) 6 months or less 3) 6-11months 4) 12 months and more
291. Did you use contraceptive methods? 1) Yes 2) No
292. What method of contraception did you use most? 1) Hormones (pills, injectable) 2) IUD 3) Condoms 4) Natural way
293. Are you divorced? 1) Yes 2) No
294. Have you had ectopic pregnancy? 1) No 2) 1 3) 2 4) 3 and more
295. Have you had hysterectomy? 1. Yes 2. No
296. Your age at the time of surgery: 1) Less than 30 years 2) 30-39 years 3) 40-49 years 4) 50 years and above
297. Did you have surgery for removing the ovaries? 1. Yes 2. No
298. Your age at the time of surgery: 1) Less than 30 years 2) 30-39 years 3) 40-49 years 4) 50 years and above
299. Have you had a tubal ligation to prevent pregnancy? 1. Yes 2. No
300. Your age at the time of surgery: 1) Less than 30 years 2) 30-39 years 3) 40-49 years 4) 50 years and above
